# Ensuring fairness in assessment in health professions education: rapid analysis tools to detect differential item functioning across groups

**DOI:** 10.5116/ijme.6694.de69

**Published:** 2024-07-23

**Authors:** Mohsen Tavakol, Claire Stewart, Claire C. Sharpe

**Affiliations:** 1 Education Centre, School of Medicine, Faculty of Medicine and Health Sciences, The University of Nottingham, UK

**Keywords:** Fairness in assessment, health professions education, differential item functioning, DIF tools

## Introduction

In high-stakes assessments, ensuring that test scores are both reliable and valid is essential, but it is equally important that they are fair. Bias in testing arises when the exam demands knowledge outside of what it is meant to assess, rendering the scores less accurate for some groups.^1^ The concept of fairness varies but generally focuses on whether assessments predict abilities equally across different groups regardless of student characteristics. The Standards for Educational and Psychological Testing emphasise the importance of fairness and inclusivity in test design and usage by suggesting minimising construct-irrelevant barriers and recording how assessment providers address potential biases during the test development process to ensure the test is fair for all intended groups of students. The Standard calls for a thorough, transparent test development and implementation process to ensure fairness and accuracy for all students, regardless of their backgrounds.^2^ With these foundational principles in place, our focus in this study is on assessments in medical education, including objective tests and OSCEs, where applying these standards is critical, even if challenging to ensure that future healthcare professionals are evaluated with fairness. Despite the critical importance of fairness in assessment, current practices in health professions education often do not focus on developing user-friendly tools to rapidly obtain results to ensure the accuracy and fairness of assessments.

In light of these foundational principles, this paper aims to enhance fairness in assessments across diverse groups by presenting innovative tools that allow rapid results analysis without requiring expertise in commercial (e.g., SPSS) or non-commercial (e.g., R language) software and statistical knowledge. There is a significant gap in the availability of accessible tools that allow for rapid analysis and immediate adjustment in cases of bias presence. Our tools directly address this critical gap by offering an efficient solution for identifying and correcting assessment biases promoting fair educational outcomes for all students. By applying interventions that are supported by research (e.g., training programs for examiners in OSCEs on unbiased ratings, moderating biased scores or reviewing and calibrating assessment tools) to effectively tackle the common and special causes of disparities in assessments, these tools help educational institutions ensure that every student has the opportunity to achieve their highest potential, regardless of background or other characteristics. Each tool includes detailed explanations of key points to ensure clarity and ease of use.

In medical education, assessments are broadly categorised into two main types: objective tests or selected-response test formats and Objective Structured Clinical Examinations (OSCEs). Objective tests, such as single-best answer (SBA) questions, aim to minimise the involvement of human judgment and subjective interpretation of scores. They are structured to provide objective measurement of student performance (objective scoring), relying on clear, correct answers that assessors can evaluate without needing to interpret responses or apply personal judgment, thus reducing potential bias.^3^ However, these formats can inadvertently introduce biases that compromise the fairness and validity of assessments. For example, cultural bias occurs when assessment content or context favours certain groups over others based on their cultural background or experiences. Language bias is another common issue, where the phrasing of assessment questions or the language used may advantage or disadvantage students based on their linguistic proficiency or familiarity with specific dialects or idioms.

On the other hand, clinical skills assessments in the form of OSCEs represent a form of subjective assessment. OSCEs involve students moving through various stations to engage in simulated clinical scenarios. Examiners assess student performance using a checklist or domain-specific checklist to evaluate specific skills alongside a global rating scale that assesses students' overall performance irrespective of the checklist scores. This global rating is often used to determine the passing mark for each station using the borderline regression method. As assessments on OSCES are based on direct observation and judgment by the examiners, they inherently involve a degree of subjective interpretation. Such subjectivity can make the scoring prone to the examiners' individual perceptions, opinions or biases, potentially compromising the fairness of the assessment outcomes and the validity of the OSCE. We use two established approaches to detect item /station bias to make assessments accurate and fair in medical education assessment: Differential Item Functioning (DIF) and Variance Components (VCs) within Generalizability Theory.

### Differential Item Functioning (DIF)

Sometimes, certain aspects of assessment questions not related to what they are intended to measure or how the test is administered can impact scores obtained by people from different groups. DIF is a way to examine if a test question is fair for everyone. Imagine an assessment question that students from two distinct groups (females and males) with equal ability attempt the item. If one group consistently finds the question easier or more challenging than the other, then the question might not be fair. DIF identifies such questions to ensure the test treats all students equally, regardless of their background or personal characteristics. The importance of DIF is emphasised in the Standards, which state, "DIF is said to occur when equally able test takers differ in their probabilities of answering a test item correctly as a function of group membership."^2^ Assessment providers often use DIF to identify and address these potential biases. DIF analysis helps pinpoint assessment questions that perform differently for subgroups of students who have similar levels of ability, thus indicating possible biases in the assessment items.

DIF analysis can be applied to OSCEs, even on a per-station basis. While traditionally used in the context of objective tests to identify questions that might be unfairly easier or harder for specific subgroups of students, DIF can also be helpful in OSCEs. In this setting, each station in an OSCE can be viewed as an "item" or "question," and DIF analysis can help determine if certain stations are systematically more difficult or easier for different groups of students (e.g., based on gender, ethnicity, disability), despite having comparable levels of competence. This application of DIF can help ensure that OSCE stations are fair and unbiased, providing all students an equal opportunity to demonstrate their clinical skills.

It is important to note that finding DIF in a test question does not necessarily mean the question is biased. Detecting DIF is just the first step. Further evidence and analysis are needed to understand why the DIF occurs and to confirm whether it indicates actual bias or a legitimate difference in how different groups understand or respond to an item. This additional evidence helps ensure that decisions to modify or remove items are based on a comprehensive understanding of the DIF's causes and implications.

### Assessment fairness analysis

In the context of ensuring fairness by student demographics or membership groups (e.g., gender, ethnicity), using variance components (VCs) in Generalizability Theory for OSCEs can also offer valuable insights into how different sources of variance impact the fairness of the assessment for these groups. By analysing these VCs for different membership groups, it is possible to examine if certain groups are consistently advantaged or disadvantaged by specific aspects of the OSCE. For example, if one station always has more varied results for one group compared to others, this could suggest a problem with fairness. Additionally, we use simple graphs called boxplots to help us see and compare these differences more clearly. These charts show the middle value, the range of values, and any extreme values for each group, making it easier to see how they differ. Using statistical procedures and effect size, we will deeply understand whether the differences matter or not.

### Fairness evaluation tools

We have developed six open-access tools to quickly analyse assessments using methods like DIF and variance components in Generalizability Theory. These tools are specifically created to help identify and address potential fairness issues among different student demographics.

   1.     For dichotomous data (where items are scored as either correct (1) or incorrect (0)) and when analysing binary groups like gender (Female and Male), use the Rasch model for detecting DIF by accessing: https://mt17.shinyapps.io/Raschdif/.

   2.     For multidomain groups (e.g., Asian, Mixed, Black, and White) with dichotomous items (0,1), review item measures using the Rasch model and the Item Characteristic Curve (ICC)^4^ for each group through: https://mt17.shinyapps.io/DIF_ICC/.

   3.     For multinomial groups with dichotomous items involving more than two categories, using logistic regression for DIF analysis at: https://mt17.shinyapps.io/Logesticdif/.

   4.     For additional DIF analysis tools suitable for any categorical group, supporting differential item scoring and partial credit scoring, visit: https://mt17.shinyapps.io/ORdif/. This tool is particularly useful for detecting bias in OSCEs at each station, where checklist scores, including those for different domains, may vary.

   5.     The Generalised Mantel-Haenszel (GMH) method is a relatively robust approach for identifying assessment biases, applicable to dichotomous questions and OSCEs with multiple categories of groups. However, it is not suitable for nonuniform DIF. To run DIF using the GMH approach, visit: https://mt17.shinyapps.io/GMHDIF/.

   6.     Using G theory, bias can be assessed for the membership groups effect and the interaction between examiner and group effect in OSCEs, ensuring fairness and accuracy in assessments. Understanding how group influences assessment outcomes and how examiners interact with group effects helps identify and address biases, which in turn improve the validity and reliability of the results. For analysing variance components, visit: https://mt17.shinyapps.io/var_comp/.

Now, we will take a closer look at the last tool. Examiners are nested within their groups (for example, gender or ethnicity), which is a common design in OSCEs. It should be noted that when different examiners in OSCEs assess groups, it is hard to tell if student performances are due to the students themselves or the mix of examiners and groups. So, in such studies, we do not try to separate examiner effects but look at the effects of groups and how examiners interact with groups.^5^ Therefore, in this design, where examiners are nested within groups, the sources of variation (effects) are the group and the Examiner by-group interaction. We examine the variance components for these factors separately to understand their individual impacts on student performance. This design uses random effects to account for factors introducing variability into OSCEs. We measure how variability in these factors influences student performance. Demographic factors like gender and ethnicity are considered random effects because they can vary across different groups and settings, and we want to understand and be able to generalise results across these varying groups rather than focusing on the performance of one specific demographic group.

The Group Effect as a source of variation refers to how different student groups (based on characteristics like gender, ethnicity or disability) inherently perform on assessments independent of the examiners. Such an effect captures the differences in performance attributed only to the group membership of the students. The examiner-by-group interaction highlights how examiners' perceptions and biases towards these diverse student backgrounds can influence scoring, introducing complexity in pinpointing the exact source of score variations—whether they arise from inherent student performance, examiner behaviour, or the specific dynamics of their interaction.

Alternatively, when examiners are crossed with groups, i.e., every examiner assesses students across all different groups, allowing for a thorough comparison and understanding of how different factors influence performance, the effects of the examiner are included in the study design. This tool is not suitable for use. In this situation, readers are advised to search for other software in generalizability studies that are better suited to their analysis needs.

Therefore, identifying and addressing the "awarding gap" or "attainment gap" is a complex but crucial task for promoting educational fairness. Merely focusing on the difference between top performers in a majority group (for example, white students) and top performers among minorities (for example, BAME students) may not offer a deep understanding of student performance. Using quantitative (e.g., statistical analysis, DIF) and qualitative (e.g., student feedback) data to an item or station level to grasp the full context and consider factors like test reliability, validity, and item analysis will help us avoid misinterpretations.

## Conclusions

Ensuring fairness in medical education assessments, including objective tests and OSCEs, is crucial for the fair evaluation of future healthcare professionals. This requires us to minimise biases such as cultural and language biases in objective tests and subjective biases in OSCEs. When we assess and evaluate student performance, we must ensure that student demographics do not affect student performance and that scores are entirely fair, with no student being advantaged or disadvantaged. If quantitative results indicate that an item or station favours a particular group, experts should review it and reach a consensus on whether or not to adjust it. Analysing each assessment question or individual station can be time-consuming and is typically handled by measurement specialists. We have developed six tools, using the Rasch model, logistic and ordinal regression, item characteristic curves (ICC), Mantel-Haenszel and generalizability theory, that enable assessment providers to garner results and quickly adjust the scores. These tools allow users to quickly detect DIF by selecting items based on group type (dichotomous or multinomial) and applying differential item scoring with different approaches. This will help improve the accuracy and fairness of the exam, especially if a particular group is at risk of being disadvantaged. In addition, emphasising transparency in test development and implementation, alongside using both quantitative and qualitative data, supports the achievement of educational fairness and the success of all students, irrespective of their backgrounds.

### Disclaimer

The authors declare that they are not responsible for the outcomes of using these tools. Users should ensure the accuracy of their data inputs. If you encounter any issues or errors, please first ensure that the file you have uploaded is correct, paying particular attention to the accuracy of the specified heading columns as requested. Please contact the corresponding author for further assistance if errors persist after this verification.

### Conflicts of Interest

The authors declare they have no conflicts of interest.
